# Temporal, seasonal and weather effects on cycle volume: an ecological study

**DOI:** 10.1186/1476-069X-11-12

**Published:** 2012-03-08

**Authors:** Sandar Tin Tin, Alistair Woodward, Elizabeth Robinson, Shanthi Ameratunga

**Affiliations:** 1 Section of Epidemiology and Biostatistics, School of Population Health, University of Auckland, Auckland, New Zealand; 2 School of Population Health, University of Auckland, Auckland, New Zealand

**Keywords:** Bicycling, Seasons, Weather, Temperature, Rain, Wind, Sunlight, New Zealand

## Abstract

**Background:**

Cycling has the potential to provide health, environmental and economic benefits but the level of cycling is very low in New Zealand and many other countries. Adverse weather is often cited as a reason why people do not cycle. This study investigated temporal and seasonal variability in cycle volume and its association with weather in Auckland, New Zealand's largest city.

**Methods:**

Two datasets were used: automated cycle count data collected on Tamaki Drive in Auckland by using ZELT Inductive Loop Eco-counters and weather data (gust speed, rain, temperature, sunshine duration) available online from the National Climate Database. Analyses were undertaken using data collected over one year (1 January to 31 December 2009). Normalised cycle volumes were used in correlation and regression analyses to accommodate differences by hour of the day and day of the week and holiday.

**Results:**

In 2009, 220,043 bicycles were recorded at the site. There were significant differences in mean hourly cycle volumes by hour of the day, day type and month of the year (*p *< 0.0001). All weather variables significantly influenced hourly and daily cycle volumes (*p *< 0.0001). The cycle volume increased by 3.2% (hourly) and 2.6% (daily) for 1°C increase in temperature but decreased by 10.6% (hourly) and 1.5% (daily) for 1 mm increase in rainfall and by 1.4% (hourly) and 0.9% (daily) for 1 km/h increase in gust speed. The volume was 26.2% higher in an hour with sunshine compared with no sunshine, and increased by 2.5% for one hour increase in sunshine each day.

**Conclusions:**

There are temporal and seasonal variations in cycle volume in Auckland and weather significantly influences hour-to-hour and day-to-day variations in cycle volume. Our findings will help inform future cycling promotion activities in Auckland.

## Background

It is widely acknowledged that physical activity provides substantial health benefits by delaying premature deaths [1], lowering the risk of a range of health conditions, notably cardiovascular diseases [2] and some forms of cancer [3], and enhancing emotional health [4]. Such health benefits could be achieved even with half the recommended amount of physical activity, i.e., 15 min per day for 6 days a week [5]. However, one in ten New Zealand adults are not active for at least 30 minutes a week [6,7] and one in three secondary school students are not active for at least 20 minutes on three occasions a week [8].

Cycling either for recreation or for transport plays an important role in increasing physical activity levels and is suitable for people of all ages, gender and backgrounds. In addition to its proven health benefits [9-12], cycle commuting may enhance social cohesion, community liveability and transport equity [13,14], improve safety to all road users [15], save fuel and reduce motor vehicle emissions [16]. In New Zealand, road cycling was ranked as the fifth most popular sport and recreation activity but only one-fifth of adults reported engaging in such activity at least once over twelve months [17]. Moreover, cycling for transport has declined over the past two decades [18,19] and accounts for only 1% of total time travelled [20] - a much lower level than in many European countries [21].

Given the co-benefits of active transport and low levels of cycling in New Zealand, factors that could influence cycling behaviour are worthy of investigation. Adverse weather is often cited as a reason why people do not cycle [22-24]. A previous New Zealand survey indicates lower likelihood of cycle commuting on a cold and wet day than on a warm and dry day [25]. Overseas studies have quantified the effects of season and weather on cycling [26-39]. However, some studies used aggregate data to compare the levels of cycling across cities or regions with different weather patterns [27,32-34] and it was not possible to assess the effect of weather changes on cycling in a particular location. Others used daily data and did not account for a lower level of cycling and possibly more severe weather during night time [26,28-31,35]. Moreover, many used manual cycle counts [28,29] or self-reported survey data [26,27,31-34,37-39] collected over a specified period, adding more inaccuracy.

We therefore investigated temporal and seasonal variability in cycle volume and the effect of weather on hour-to-hour and day-to-day variations in cycle volume in Auckland, New Zealand's largest city, using a year of continuous automated cycle count data.

## Methods

Auckland is situated on an isthmus in the north of the North Island and has four defined seasons which are the inverse of those experienced in the northern hemisphere - summer is from December to February; autumn from March to May; winter from June to August; and spring from September to November. The city has a mild subtropical climate with a mean temperature of 14.6°C, 955 mm rainfall and 2176 sunshine hours in 2009 (the study year) [40]. Despite this, Auckland is reported to have a very low level of cycling [41-43]. Between 2008 and 2010, use of a bicycle represented only 1% of all time spent travelling whereas driver and passenger trips accounted for 80% [41].

### Data sources

Two datasets were used for this analysis: automated cycle count data collected by Integrated Traffic Solutions Ltd (ITS) [44] and the National Climate Database maintained by National Institute of Water & Atmospheric Research (NIWA) [45]. Analyses were undertaken using data collected over one year from 1 January to 31 December 2009.

#### Cycle count data

**Figure 1:**
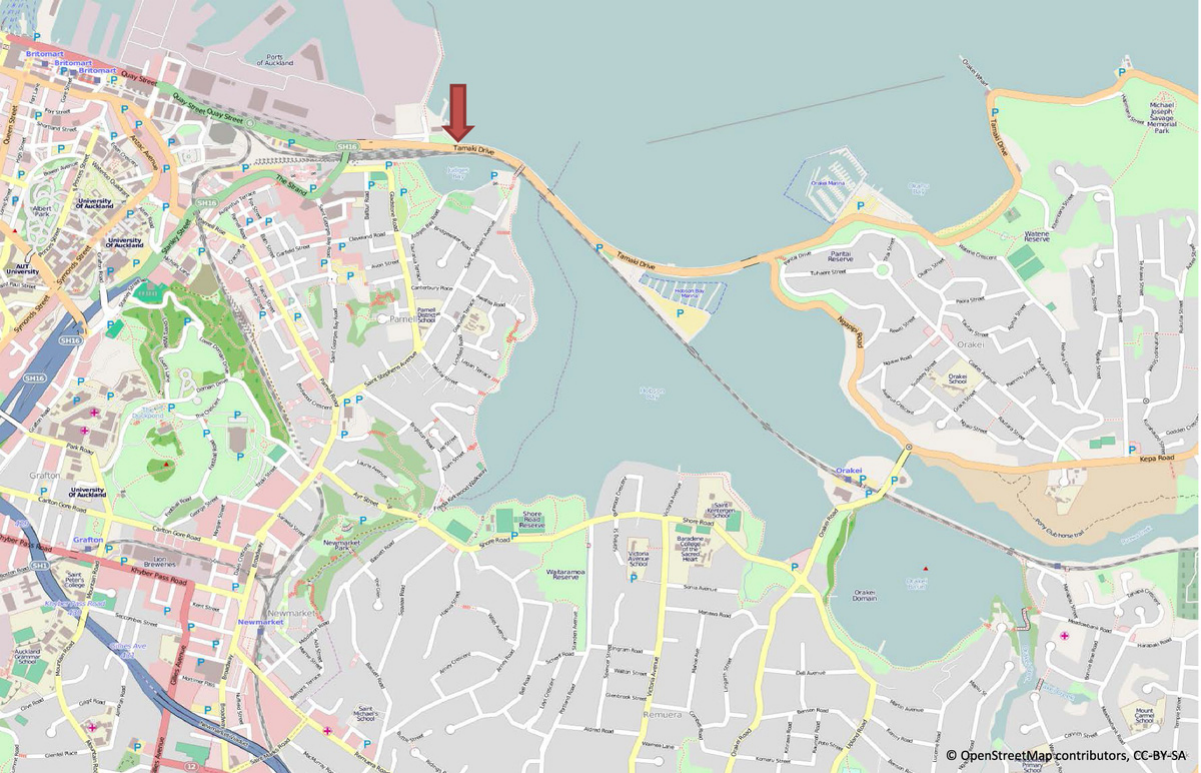
**Location of cycle counters**. Red downwards arrow symbol indicates location of cycle counters on Tamaki Drive.

Continuous automated cycle counting has been undertaken by ITS at a single site on Tamaki Drive in Auckland since December 2008 (Figure 1). Tamaki Drive runs ten kilometres along the coastline from the central business district to St Heliers Bay and is a busy cycling route for both recreational and commuting purposes. ZELT Inductive Loop Eco-counters were used to record bicycle counts. The site incorporated four inductive loops were inserted under the surface on the side of the road where traffic flows out of the city, that is, the upper side of the road in Figure 1. Note that traffic keeps to the left side of the road in New Zealand. Two loops were inserted on the on-road bicycle lane (adjacent to but not separated from motorised traffic) and two on the off-road shared bicycle and pedestrian path (separated from motorised traffic). Cyclists travel in the same direction as motorised traffic on the bicycle lane whereas they often travel in both directions on the shared path.

Each time a bicycle goes over the loop, the system detects the electromagnetic signature of the two wheels and registers a count. The on-road system can distinguish between motorised traffic and bicycles and the off-road system can distinguish individual cyclists while also detecting groups of cyclists. The system has accuracy ranging between 94% and 98%, with the off-road counters being more sensitive and accurate to groups than the on-road counters, which are made to focus on detecting cyclists in mixed traffic conditions (personal communications, Kyle Donegan (ITS)). As the direction of travel along the off-road path was not monitored, total cycle volume regardless of direction was used in this analysis.

#### Weather data

Weather variables of interest for this analysis include: maximum gust speed, rain, maximum temperature and duration of sunshine. The data were obtained from the National Climate Database which holds data from about 6500 climate stations which have been operating for various periods since 1850. Over 600 stations are currently operating. Weather conditions at the cycle counting site were approximated by the hourly weather conditions recorded in the closest stations. Gust speed, rain and temperature data were obtained from a station situated 2.5 km from the cycle counting site and duration of sunshine data, which were not available in closer stations, were obtained from a station located about 12.5 km from the cycle counting site.

### Data analysis

Maximum gust speed, total rain, maximum temperature and total duration of sunshine on a daily basis were computed from the hourly weather data. Summary information (mean, standard deviation and range) on hourly and daily weather conditions were presented for the whole year 2009 and by season.

The variability in mean hourly cycle volumes by hour of the day, by day of the week and holiday, and by month of the year was explored using Analysis of Variance (ANOVA). There were eleven holidays in 2009, and for all those holidays adjacent weekends were also considered as a holiday, resulting in a total of 25 holidays.

To assess the effects of weather on hourly and daily cycle volumes while controlling for differences by hour of the day and day of the week and holiday, cycle volumes were normalised by using the equations [46]:

normalised hourly cycle volume=hourly cycle volume/hourly mean for day type

where the hourly mean was calculated for the given hour of the day for the given day type (Sunday to Saturday or holiday).

normalised daily cycle volume=daily cycle volume/daily mean for day type

where the daily mean was calculated for the given day type (Sunday to Saturday or holiday).

The relationships between weather variables of interest and cycle volume were assessed by the Spearman's Rank-Order Correlation test and linear regression models. For the hourly data, analyses were restricted to the period between 6:00 am and 8:00 pm due to the very low cycle volume at night. All weather variables found to have significant relationships with cycle volume (*p *< 0.05) were included in multivariate models. The term "temperature squared" was used in the models to check if the relationship between temperature and cycle volume was linear or quadratic. Multicollinearity between the variables was assessed by Variance Inflation Factors (VIFs). A Newey-West estimator was used to account for heteroskedasticity and autocorrelation. Subgroup analyses were undertaken by day type (weekdays or weekends and holidays) and season. SAS (release 9.1, SAS Institute Inc., Cary, NC) was used for all analyses.

## Results

### Cycle volume

In 2009, a total of 220,043 bicycles were recorded at the site - 94,684 bicycles on lane (on-road bicycle lane) and 125,359 bicycles on path (off-road shared path).

### Weather

Hourly and daily weather data are summarised in Table 1. The site was considered windy with an average gust speed of 36.6 km/h each day. The maximum gust speed was 79.6 km/h in 2009. It experienced an average rainfall of 2.7 mm per day with more rain in winter (3.7 mm per day) than in other seasons. The maximum daily temperature ranged from 8.1°C to 28.1°C with an average of 23.2°C in summer and 14.6°C in winter. The site received six hours of sunshine each day - 7.6 hours in summer and 4.8 hours in winter.

**Table 1:** Weather variables of interest

	2009			Summer			Autumn			Winter			Spring		
	**Mean (SD)**	**Min**	**Max**	**Mean (SD)**	**Min**	**Max**	**Mean (SD)**	**Min**	**Max**	**Mean (SD)**	**Min**	**Max**	**Mean (SD)**	**Min**	**Max**

***Hourly weather data (6:00 am - 8:00 pm)***															

Maximum gust speed (km/h)	25.9 (10.5)	2.9	79.6	26.2 (9.3)	3.2	61.2	24.6 (10.9)	4.0	79.6	24.9 (11.8)	3.6	67.0	27.9 (9.6)	2.9	55.4

Rain (mm)	0.1 (0.6)	0	14.8	0.1 (0.7)	0	13.8	0.1 (0.5)	0	7.8	0.1 (0.6)	0.0	10.6	0.1 (0.7)	0.0	14.8

Maximum temperature (°C)	16.4 (4.2)	3.4	28.1	21.1 (2.7)	13.4	28.1	17.1 (3.6)	5.4	25.5	12.5 (2.5)	3.4	18.4	15.3 (2.5)	4.8	22.3

Sunshine duration (hour)	0.4 (0.4)	0.0	1.0	0.5 (0.4)	0.0	1.0	0.4 (0.4)	0.0	1.0	0.3 (0.4)	0.0	1.0	0.4 (0.4)	0.0	1.0

***Daily weather data***															

Maximum gust speed (km/h)	36.6 (9.8)	15.1	79.6	35.6 (8.6)	20.2	68.0	35.8 (10.0)	16.9	79.6	36.7 (11.8)	15.1	67.0	38.2 (8.3)	17.6	55.4

Rain (mm)	2.7 (6.0)	0.0	45.6	2.5 (7.5)	0.0	45.6	2.3 (4.8)	0.0	25.6	3.7 (6.2)	0.0	31.6	2.3 (5.3)	0.0	36.2

Maximum temperature (°C)	18.5 (3.9)	8.1	28.1	23.2 (2.4)	15.6	28.1	19.1 (3.0)	12.2	25.5	14.6 (1.7)	10.6	18.4	17.3 (2.0)	8.1	22.3

Total sunshine hours	6.0 (3.9)	0.0	13.8	7.6 (4.3)	0.0	13.8	5.9 (3.3)	0.0	11.4	4.8 (3.3)	0.0	10.2	5.6 (4.0)	0.0	12.6

### Temporal and seasonal effects on cycle volume

**Figure 2:**
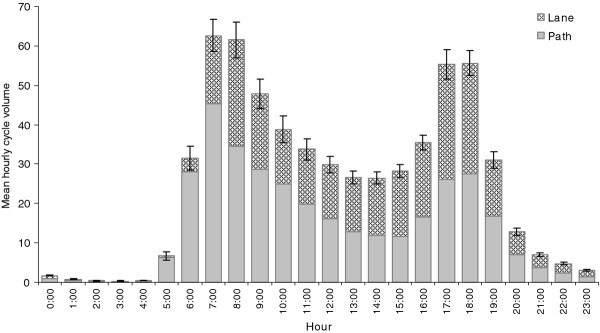
**Mean hourly cycle volume by hour**. Error bars represent 95% confidence intervals.

**Figure 3:**
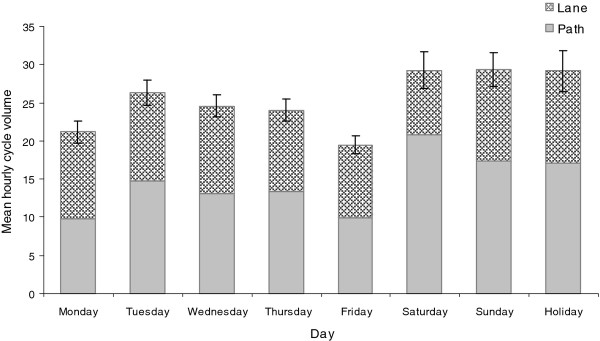
**Mean hourly cycle volume by day**. Error bars represent 95% confidence intervals.

**Figure 4:**
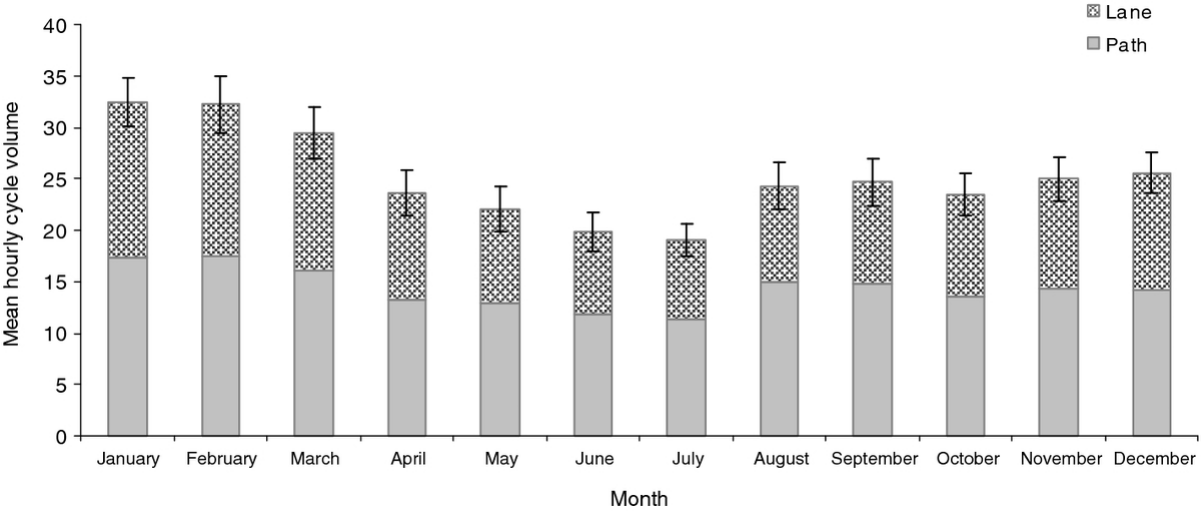
**Mean hourly cycle volume by month**. Error bars represent 95% confidence intervals.

There were significant differences (*p *< 0.0001) in mean hourly cycle volumes either on path or on lane by hour of the day (Figure 2), day of the week and holiday (Figure 3) and month of the year (Figure 4). The peak cycle volume occurred between 7:00 and 8:00 am in the morning and between 5:00 and 6:00 pm in the evening. The volume was lower on weekdays compared to weekends and holidays. The highest volume was observed in January and February, the hottest months of the year, and the lowest volume in July, the coldest month.

### Effects of weather conditions on cycle volume

Significant correlations were observed between all weather variables of interest and hourly and daily cycle volumes, either on lane or on path (*p *< 0.0001) (Table 2). Gust speed and rain were negatively correlated with cycle volumes while temperature and sunshine duration were positively correlated. The correlations were stronger for the daily data compared to the hourly data.

**Table 2:** Spearman rank correlation between weather variables and normalised cycle volume

	Path		Lane		Total	
	***ρ ***	***p*-value**	***ρ ***	***p*-value**	***ρ ***	***p*-value**

***Hourly cycle volume (6:00 am - 8:00 pm)***						

Maximum gust speed (km/h)	-0.21	< 0.0001	-0.14	< 0.0001	-0.22	< 0.0001

Rain (mm) in an hour	-0.32	< 0.0001	-0.30	< 0.0001	-0.35	< 0.0001

Maximum temperature (°C)	0.21	< 0.0001	0.36	< 0.0001	0.30	< 0.0001

Hour with sunshine	0.28	< 0.0001	0.35	< 0.0001	0.34	< 0.0001

***Daily cycle volume ***						

Maximum gust speed (km/h)	-0.52	< 0.0001	-0.40	< 0.0001	-0.48	< 0.0001

Rain (mm) in a day	-0.63	< 0.0001	-0.64	< 0.0001	-0.65	< 0.0001

Maximum temperature (°C)	0.40	< 0.0001	0.54	< 0.0001	0.49	< 0.0001

Sunshine hours in a day	0.53	< 0.0001	0.61	< 0.0001	0.58	< 0.0001

Estimates for the weather variables, standard errors and significance levels of the multivariate linear regression models are shown in Table 3. Temperature squared was excluded from the final models as its coefficient was not significant. All weather variables significantly influenced both hourly and daily cycle volumes. The hourly cycle volume (total) increased by 3.2% for 1°C increase in temperature but decreased by 1.4% for 1 km/h increase in gust speed and by 10.6% for 1 mm increase in rainfall during that hour. The volume was 26.2% higher in an hour with sunshine compared with no sunshine. Weather variables accounted for a greater variance in daily cycle volume (56%) compared to the hourly cycle volume (23%) although a weaker effect was observed on daily cycle volume. The daily cycle volume (total) increased by 2.6% for 1°C increase in temperature but decreased by 0.9% for 1 km/h increase in gust speed and by 1.5% for 1 mm increase in rainfall during that day. The volume increased by 2.5% for one hour increase in sunshine each day. The effect of temperature and sunshine duration was stronger on the cycle volume on lane than on path.

**Table 3:** Multivariate linear regression models for normalised cycle volume

	Path			Lane			Total		
	**Estimate**	**SE**	***p*-value**	**Estimate**	**SE**	***p*-value**	**Estimate**	**SE**	***p*-value**

***Hourly cycle volume (6:00 am to 8:00 pm)***									

Intercept	0.885	0.046		0.475	0.057		0.722	0.042	

Maximum gust speed (km/h)	-0.014	0.001	< 0.0001	-0.014	0.001	< 0.0001	-0.014	0.001	< 0.0001

Rain (mm) in an hour	-0.106	0.015	< 0.0001	-0.101	0.014	< 0.0001	-0.106	0.014	< 0.0001

Maximum temperature (°C)	0.023	0.003	< 0.0001	0.048	0.004	< 0.0001	0.032	0.003	< 0.0001

Hour with sunshine	0.247	0.027	< 0.0001	0.268	0.031	< 0.0001	0.262	0.026	< 0.0001

	**R^2 ^= 0.16**			**R^2 ^= 0.19**			**R^2 ^= 0.23**		

***Daily cycle volume***									

Intercept	0.960	0.096		0.414	0.103		0.730	0.091	

Maximum gust speed (km/h)	-0.011	0.001	< 0.0001	-0.007	0.001	< 0.0001	-0.009	0.001	< 0.0001

Rain (mm) in a day	-0.014	0.002	< 0.0001	-0.016	0.003	< 0.0001	-0.015	0.002	< 0.0001

Maximum temperature (°C)	0.019	0.004	< 0.0001	0.037	0.005	< 0.0001	0.026	0.004	< 0.0001

Sunshine hours in a day	0.020	0.004	< 0.0001	0.033	0.004	< 0.0001	0.025	0.004	< 0.0001

	**R^2 ^= 0.49**			**R^2 ^= 0.57**			**R^2 ^= 0.56**		

In subgroup analyses by day type (weekdays or weekends and holidays), similar findings were observed except that the relationship between temperature and cycle volume was not significant during weekends and holidays (Additional files 1 and 2).

In subgroup analyses by season, there was no significant effect of temperature on hourly cycle volume during summer and spring and on daily cycle volumes during summer and winter (Additional files 1 and 2). Sunshine duration did not predict daily cycle volume on path during summer.

## Discussion

The cycle volume on Tamaki Drive in Auckland City varied by hour of the day, day of the week and month of the year. Although Auckland has a subtropical climate without extreme weather conditions, the findings suggest a significant impact of weather (gust speed, rain, temperature and sunshine duration) on both hourly and daily cycle volumes. Warm and sunny weather increases the cycle volume while rainy and windy weather reduces it. Overall, the selected weather variables accounted for 23% of the variance in hourly cycle volume and 56% of the variance in daily cycle volume.

A major strength of the present study is the use of a year's worth of continuous automated cycle count data to quantify the effect of weather on both hourly and daily cycle volumes. However, some limitations need attention. As the data were collected from only one site with a reasonably narrow range of weather conditions, the findings may not be generalisable to locations with different climate and weather patterns or different cycling environments. The accuracy of the ZELT Inductive Loop Eco-counters may also be limited. While the counters used at the site have the accuracy (sensitivity) ranging between 94% and 98%, a previous New Zealand study estimated an accuracy of 80% off-road and 90% on-road [47]. Recent research from the US reported a 3% undercount on the separate path and a 4% overcount on the shared roadway [48]. It is possible that an individual bicycle may be undetected if it is made from non-metallic materials such as carbon fibre [49] or if groups of cyclists are not counted effectively [47]. In contrast, double counting may occur as two loops are used at the site [47].

Nevertheless, our findings are consistent with the evidence gained from previous research reporting temporal and seasonal variations in cycle volume [28,30,31,35,38,39]. The seasonality differed by demographic characteristics in recent studies. A US study found a greater sensitivity in children and adolescents, women, non-Caucasians, persons with low income and persons living in urbanised areas [38]. A Norwegian study reported that children of parents with higher education were more likely to cycle to school in fall and spring and less likely to cycle (but more likely to walk) in winter [39]. However, we were not able to investigate this due to lack of demographic information in our data.

Weather conditions, mainly temperature and rainfall, significantly influenced cycle flows in previous studies [26,28-31,33-37] although no obvious association was reported in some studies [27,32]. It is likely that a quadratic relationship exists between cycle volume and temperature suggesting a lower volume at very cold and very hot temperatures [31]. Although we found a significant effect of temperature on both hourly and daily cycle volumes, particularly on lane, we did not observe the quadratic pattern possibly due to the lack of temperature extremes at the study site. The narrow temperature range may also be a reason why temperature lost its significant effect in subgroup analyses by season.

Although temperature was found to be a stronger predictor than rainfall in some studies [28-30,36], we found that rainfall significantly reduced hourly cycle volume throughout the year - ranging from an 8% reduction during spring to a 13% reduction during summer and autumn with a 1 mm increase in rainfall. This confirms the common perception that rain is a major deterrent to cycling [29,50] and is consistent with a previous review showing the largest correlation between precipitation and physical activity [51].

Other weather conditions have received less attention in published studies. Some studies reported the negative impact of wind speed on cycle volume [29,35-37] but others did not [32]. We were not surprised to see a significant negative association between gust speed and cycle volume in our study given that the study site is often windy. Although the effect of sunshine was not significant in a previous study [36], we found that sunshine hours strongly influenced cycle volume particularly in winter and spring. The on-lane cycle volume was 35% higher in the sunshine hour compared to the hour without sun during winter.

The seasonal and weather effects appear to be stronger on recreational cycling compared to utilitarian cycling [26,31,35]. However, we were not able to differentiate utilitarian vs. recreational cyclists from our cycle count data. One might assume that commuting cyclists would use more direct on-road lanes and recreational cyclists would use more enjoyable off-road paths. However, a.m. and p.m. peaks suggest that the majority of cyclists were commuting on both on-road lanes and off-road paths, while the high cycle volume at weekends and on holidays shows that there were a reasonable number of recreational cyclists on both cycleways. We observed similar weather effects on cycling during weekdays and weekends or holidays except that the effect of temperature was not significant in the latter.

Our findings may help develop temporal, seasonal and weather corrections to standardise cycle flows in future surveys which are mostly conducted at a specified time during the year. The findings also suggest that season and weather may need to be taken into account in future policies and interventions aiming to promote cycling in Auckland. Simple remedies include use of protective gears, for example, wearing a rain jacket and having a shower afterwards. Given a favourable trend in cycling as a recreational activity in New Zealand [17], provision of shower and change facilities and secure bicycle storage at destinations such as workplaces, universities, and schools may encourage people to move from recreational to commuter cycling [52]. Provision of better public transport infrastructure integrated with cycling may also facilitate utility cycling in any weather. Currently public transport represents only 5% of all time spent travelling in Auckland [41]. While the majority of bicycle related injuries occur in good weather when people cycle more, proper maintenance and care of bicycles, public roads and cycleways may prevent crashes which are likely to be severe in bad weather [53,54].

However, the effects of weather should not be overemphasised. In fact, Auckland gets more annual sunshine and less temperature extremes compared to European cities, yet the city has a much lower level of cycling [21,55]. For example, cycling accounts for 38% of all vehicle trips in Amsterdam (Netherlands) [56] (cf. 1% in Auckland [41]). It is likely that a relatively high rainfall in Auckland discourages people from cycling; however, the estimated chance of getting wet while cycling is only once every fortnight if the trip is less than an hour [57]. Although Auckland's hilly roads could be another barrier, cycling is fairly prevalent in other hilly towns such as Trondheim (Norway) [55] and San Francisco (US) [58].

## Conclusions

There are temporal and seasonal variations in cycle volume on Tamaki Drive in Auckland. The selected weather conditions significantly influence both hour-to-hour and day-to-day variations in cycle volumes - temperature and sunshine duration have a positive effect and rainfall and gust speed have a negative effect. Our findings will help inform future policies and interventions aiming to make Auckland a "cyclable" city throughout the year.

## Abbreviations

ITS: Integrated Traffic Solutions Ltd; km/h: kilometres per hour; NIWA: National Institute of Water and Atmospheric Research.

## Competing interests

The authors declare that they have no competing interests.

## Authors' contributions

STT has contributed to acquisition, analysis and interpretation of data and drafting the manuscript. AW, ER and SA have contributed to interpretation of data and revising the manuscript critically. All authors have given final approval of the version to be published.

## Supplementary Material

Additional file 1 **Multivariate linear regression models for normalised hourly cycle volume (6:00 am - 8:00 pm) by day types and season**. Click here for file

Additional file 2 **Multivariate linear regression models for normalised daily cycle volume by day types and season**. Click here for file
