# Supplementary material for: Temporal, seasonal and weather effects on cycle volume: an ecological study
Source: Environ Health. 2012 Mar 8;11:12. doi: 10.1186/1476-069X-11-12 (PMC3368741; doi:10.1186/1476-069X-11-12)
Supplement: Additional file 1 — Multivariate linear regression models for normalised hourly cycle volume (6:00 am - 8:00 pm) by day types and season. [file 1476-069X-11-12-S1.DOCX]

### Additional file 1 – Multivariate linear regression models for normalised hourly cycle volume (6:00 am – 8:00 pm) by day types and season

|  | **Path** | | | **Lane** | | | **Total** | | |
| --- | --- | --- | --- | --- | --- | --- | --- | --- | --- |
|  | **Estimate** | **SE** | **p-value** | **Estimate** | **SE** | **p-value** | **Estimate** | **SE** | **p-value** |
| ***Weekdays*** |  |  |  |  |  |  |  |  |  |
| Intercept | 0.860 | 0.052 |  | 0.361 | 0.058 |  | 0.681 | 0.048 |  |
| Maximum gust speed (km/h) | -0.014 | 0.001 | <0.0001 | -0.014 | 0.001 | <0.0001 | -0.014 | 0.001 | <0.0001 |
| Rain (mm) in an hour | -0.112 | 0.018 | <0.0001 | -0.104 | 0.016 | <0.0001 | -0.111 | 0.017 | <0.0001 |
| Maximum temperature (ºC) | 0.025 | 0.003 | <0.0001 | 0.055 | 0.004 | <0.0001 | 0.035 | 0.003 | <0.0001 |
| Hour with sunshine | 0.250 | 0.030 | <0.0001 | 0.261 | 0.035 | <0.0001 | 0.254 | 0.028 | <0.0001 |
|  | *R^2^=0.17* | | | *R^2^=0.21* | | | *R^2^=0.24* | | |
| ***Weekends and holidays*** |  |  |  |  |  |  |  |  |  |
| Intercept | 0.934 | 0.093 |  | 0.708 | 0.140 |  | 0.807 | 0.085 |  |
| Maximum gust speed (km/h) | -0.014 | 0.002 | <0.0001 | -0.015 | 0.002 | <0.0001 | -0.014 | 0.002 | <0.0001 |
| Rain (mm) in an hour | -0.095 | 0.025 | 0.0002 | -0.101 | 0.030 | 0.0009 | -0.096 | 0.025 | 0.0001 |
| Maximum temperature (ºC) | 0.021 | 0.006 | 0.0004 | 0.034 | 0.007 | <0.0001 | 0.027 | 0.005 | <0.0001 |
| Hour with sunshine | 0.243 | 0.053 | <0.0001 | 0.295 | 0.058 | <0.0001 | 0.284 | 0.049 | <0.0001 |
|  | *R^2^=14* | | | *R^2^=0.15* | | | *R^2^=0.21* | | |
| ***Summer (December-February)*** | |  |  |  |  |  |  |  |  |
| Intercept | 1.278 | 0.195 |  | 1.252 | 0.235 |  | 1.153 | 0.180 |  |
| Maximum gust speed (km/h) | -0.017 | 0.002 | <0.0001 | -0.015 | 0.003 | <0.0001 | -0.014 | 0.002 | <0.0001 |
| Rain (mm) in an hour | -0.128 | 0.029 | <0.0001 | -0.141 | 0.032 | <0.0001 | -0.130 | 0.028 | <0.0001 |
| Maximum temperature (ºC) | 0.011 | 0.009 | 0.2 | 0.017 | 0.011 | 0.1 | 0.016 | 0.008 | 0.05 |
| Hour with sunshine | 0.182 | 0.062 | 0.003 | 0.196 | 0.069 | 0.005 | 0.205 | 0.057 | 0.0003 |
|  | *R^2^=0.11* | | | *R^2^=0.08* | | | *R^2^=0.13* | | |
| ***Autumn (March-May)*** |  |  |  |  |  |  |  |  |  |
| Intercept | 0.566 | 0.105 |  | 0.368 | 0.140 |  | 0.459 | 0.092 |  |
| Maximum gust speed (km/h) | -0.013 | 0.002 | <0.0001 | -0.013 | 0.002 | <0.0001 | -0.013 | 0.002 | <0.0001 |
| Rain (mm) in an hour | -0.119 | 0.027 | <0.0001 | -0.143 | 0.032 | <0.0001 | -0.134 | 0.028 | <0.0001 |
| Maximum temperature (ºC) | 0.039 | 0.007 | <0.0001 | 0.051 | 0.009 | <0.0001 | 0.045 | 0.006 | <0.0001 |
| Hour with sunshine | 0.178 | 0.055 | 0.001 | 0.228 | 0.064 | 0.0003 | 0.211 | 0.052 | <0.0001 |
|  | *R^2^=0.15* | | | *R^2^=0.15* | | | *R^2^=0.21* | | |
| ***Winter (June-August)*** |  |  |  |  |  |  |  |  |  |
| Intercept | 0.891 | 0.086 |  | 0.663 | 0.110 |  | 0.849 | 0.075 |  |
| Maximum gust speed (km/h) | -0.011 | 0.002 | <0.0001 | -0.011 | 0.002 | <0.0001 | -0.012 | 0.001 | <0.0001 |
| Rain (mm) in an hour | -0.107 | 0.022 | <0.0001 | -0.083 | 0.017 | <0.0001 | -0.101 | 0.019 | <0.0001 |
| Maximum temperature (ºC) | 0.017 | 0.008 | 0.04 | 0.020 | 0.009 | 0.02 | 0.015 | 0.007 | 0.03 |
| Hour with sunshine | 0.280 | 0.050 | <0.0001 | 0.352 | 0.049 | <0.0001 | 0.301 | 0.045 | <0.0001 |
|  | *R^2^=0.15* | | | *R^2^=0.13* | | | *R^2^=0.20* | | |
| ***Spring (September-November)*** | |  |  |  |  |  |  |  |  |
| Intercept | 1.264 | 0.109 |  | 0.939 | 0.145 |  | 1.172 | 0.098 |  |
| Maximum gust speed (km/h) | -0.019 | 0.002 | <0.0001 | -0.016 | 0.002 | <0.0001 | -0.018 | 0.002 | <0.0001 |
| Rain (mm) in an hour | -0.087 | 0.034 | 0.0094 | -0.075 | 0.026 | 0.0038 | -0.083 | 0.030 | 0.0064 |
| Maximum temperature (ºC) | 0.007 | 0.007 | 0.4 | 0.023 | 0.008 | 0.005 | 0.011 | 0.006 | 0.08 |
| Hour with sunshine | 0.335 | 0.039 | <0.0001 | 0.337 | 0.046 | <0.0001 | 0.341 | 0.037 | <0.0001 |
|  | *R^2^=0.19* | | | *R^2^=0.13* | | | *R^2^=0.23* | | |
