# Supplementary material for: Temporal, seasonal and weather effects on cycle volume: an ecological study
Source: Environ Health. 2012 Mar 8;11:12. doi: 10.1186/1476-069X-11-12 (PMC3368741; doi:10.1186/1476-069X-11-12)
Supplement: Additional file 2 — Multivariate linear regression models for normalised daily cycle volume by day types and season. [file 1476-069X-11-12-S2.DOCX]

### Additional file 2 – Multivariate linear regression models for normalised daily cycle volume by day types and season

|  | **Path** | | | **Lane** | | | **Total** | | |
| --- | --- | --- | --- | --- | --- | --- | --- | --- | --- |
|  | **Estimate** | **SE** | **p-value** | **Estimate** | **SE** | **p-value** | **Estimate** | **SE** | **p-value** |
| ***Weekdays*** |  |  |  |  |  |  |  |  |  |
| Intercept | 0.813 | 0.091 |  | 0.170 | 0.087 |  | 0.548 | 0.080 |  |
| Maximum gust speed (km/h) | -0.010 | 0.001 | <0.0001 | -0.006 | 0.001 | <0.0001 | -0.008 | 0.001 | <0.0001 |
| Rain (mm) in a day | -0.014 | 0.002 | <0.0001 | -0.015 | 0.003 | <0.0001 | -0.014 | 0.002 | <0.0001 |
| Maximum temperature (ºC) | 0.025 | 0.004 | <0.0001 | 0.049 | 0.005 | <0.0001 | 0.035 | 0.004 | <0.0001 |
| Sunshine hours in a day | 0.019 | 0.003 | <0.0001 | 0.030 | 0.004 | <0.0001 | 0.023 | 0.003 | <0.0001 |
|  | *R^2^=0.55* | | | *R^2^=0.68* | | | *R^2^=0.65* | | |
| ***Weekends and holidays*** |  |  |  |  |  |  |  |  |  |
| Intercept | 1.375 | 0.168 |  | 1.039 | 0.231 |  | 1.218 | 0.177 |  |
| Maximum gust speed (km/h) | -0.015 | 0.003 | <0.0001 | -0.012 | 0.003 | 0.0005 | -0.014 | 0.003 | <0.0001 |
| Rain (mm) in a day | -0.018 | 0.006 | 0.0021 | -0.021 | 0.006 | 0.0007 | -0.019 | 0.006 | 0.0006 |
| Maximum temperature (ºC) | 0.005 | 0.007 | 0.4 | 0.012 | 0.009 | 0.2 | 0.008 | 0.007 | 0.2 |
| Sunshine hours in a day | 0.022 | 0.008 | 0.0054 | 0.038 | 0.009 | <0.0001 | 0.029 | 0.008 | 0.0003 |
|  | *R^2^=0.43* | | | *R^2^=0.48* | | | *R^2^=0.48* | | |
| ***Summer (December-February)*** | |  |  |  |  |  |  |  |  |
| Intercept | 1.219 | 0.334 |  | 0.668 | 0.464 |  | 1.011 | 0.362 |  |
| Maximum gust speed (km/h) | -0.012 | 0.003 | 0.0002 | -0.007 | 0.004 | 0.0819 | -0.010 | 0.003 | 0.0025 |
| Rain (mm) in a day | -0.014 | 0.004 | 0.0015 | -0.016 | 0.006 | 0.0137 | -0.015 | 0.005 | 0.0043 |
| Maximum temperature (ºC) | 0.012 | 0.012 | 0.3 | 0.030 | 0.018 | 0.1 | 0.019 | 0.013 | 0.2 |
| Sunshine hours in a day | 0.013 | 0.008 | 0.1046 | 0.026 | 0.010 | 0.0133 | 0.019 | 0.008 | 0.0298 |
|  | *R^2^=0.45* | | | *R^2^=0.41* | | | *R^2^=0.46* | | |
| ***Autumn (March-May)*** |  |  |  |  |  |  |  |  |  |
| Intercept | 0.733 | 0.206 |  | 0.287 | 0.201 |  | 0.553 | 0.165 |  |
| Maximum gust speed (km/h) | -0.010 | 0.003 | 0.0003 | -0.005 | 0.002 | 0.0516 | -0.007 | 0.002 | 0.001 |
| Rain (mm) in a day | -0.016 | 0.006 | 0.0154 | -0.020 | 0.007 | 0.006 | -0.018 | 0.006 | 0.0068 |
| Maximum temperature (ºC) | 0.026 | 0.009 | 0.004 | 0.038 | 0.010 | 0.0004 | 0.031 | 0.008 | 0.0003 |
| Sunshine hours in a day | 0.023 | 0.010 | 0.0296 | 0.035 | 0.012 | 0.0036 | 0.028 | 0.011 | 0.0095 |
|  | *R^2^=0.36* | | | *R^2^=0.44* | | | *R^2^=0.44* | | |
| ***Winter (June-August)*** |  |  |  |  |  |  |  |  |  |
| Intercept | 0.852 | 0.233 |  | 0.735 | 0.251 |  | 0.803 | 0.219 |  |
| Maximum gust speed (km/h) | -0.009 | 0.002 | 0.0003 | -0.007 | 0.002 | 0.0006 | -0.008 | 0.002 | 0.0003 |
| Rain (mm) in a day | -0.014 | 0.003 | <0.0001 | -0.016 | 0.003 | <0.0001 | -0.015 | 0.003 | <0.0001 |
| Maximum temperature (ºC) | 0.023 | 0.015 | 0.1 | 0.016 | 0.015 | 0.3 | 0.020 | 0.013 | 0.1 |
| Sunshine hours in a day | 0.017 | 0.007 | 0.0263 | 0.031 | 0.008 | 0.0001 | 0.022 | 0.007 | 0.0031 |
|  | *R^2^=0.46* | | | *R^2^=0.50* | | | *R^2^=0.52* | | |
| ***Spring (September-November)*** | |  |  |  |  |  |  |  |  |
| Intercept | 1.072 | 0.165 |  | 0.749 | 0.216 |  | 0.939 | 0.172 |  |
| Maximum gust speed (km/h) | -0.016 | 0.002 | <0.0001 | -0.009 | 0.003 | 0.0007 | -0.013 | 0.002 | <0.0001 |
| Rain (mm) in a day | -0.016 | 0.002 | <0.0001 | -0.015 | 0.003 | <0.0001 | -0.016 | 0.003 | <0.0001 |
| Maximum temperature (ºC) | 0.025 | 0.009 | 0.009 | 0.022 | 0.009 | 0.01 | 0.023 | 0.008 | 0.004 |
| Sunshine hours in a day | 0.026 | 0.004 | <0.0001 | 0.037 | 0.005 | <0.0001 | 0.030 | 0.004 | <0.0001 |
|  | *R^2^=0.60* | | | *R^2^=0.54* | | | *R^2^=0.61* | | |
